# The implementation and evaluation of an athlete leadership development program with male youth ice hockey players

**DOI:** 10.3389/fpsyg.2022.648039

**Published:** 2022-10-14

**Authors:** Matthieu M. Boisvert, Todd M. Loughead, Krista J. Munroe-Chandler

**Affiliations:** Department of Kinesiology, University of Windsor, Windsor, ON, Canada

**Keywords:** applied sport psychology, group dynamics, cohesion, collective efficacy, athlete leadership, athlete leadership development

## Abstract

The purpose of the current study was to implement and evaluate an athlete leadership development program in youth boys ice hockey. The sample consisted of 14 male U17 hockey players (*M* = 16.46, *SD* = 0.78) from one team playing in a competitive hockey league. The players participated in six leadership intervention workshops over the course of the season, and completed inventories measuring athlete leadership behaviours, cohesion, and collective efficacy pre-and post-intervention. In addition, a focus group was conducted to assess the impact of the athlete leadership development program at the end of the season. Bayesian *t* tests showed that the leadership program generally helped to maintain levels of athlete leadership behaviours, cohesion, and collective efficacy pre-and post-intervention. The results of the focus group following the intervention revealed the players believed the leadership development program helped buffer against the negative effects of their on-ice performances.

## Introduction

The importance of leadership in sport is well documented (e.g., Bucci et al., 2012). In fact, effective leadership is identified as a crucial factor in achieving team success (Zaccaro et al., 2002). To date, most of the research examining leadership in sport has primarily focused on the coach, which is not surprising given the coach is responsible for making decisions with respect to team matters such as strategy, tactics, and team personnel (Loughead et al., 2006). Despite Gould’s statement in Gould et al. (1987), suggesting that coaches consider athlete leadership as an important component for effective team performance, only recently has athlete leadership in sport teams received attention (Loughead, 2017).

Athlete leadership is defined as “an athlete occupying a formal or informal role within a team who influences team members to achieve a common goal” (Loughead et al., 2006, p. 144). The above definition highlights two types of leadership roles that are shared within sport teams. First, formal athlete leaders are those who are assigned to their leadership role by the coach or through team selection (e.g., captain, assistant captain). Second, informal athlete leaders emerge based on their interactions with other teammates (e.g., veteran players). Crozier et al. (2013) examined what athletes considered to be the ideal number of athlete leaders on a team as well as the benefits of having athlete leaders. Athletes indicated that 85% of a team’s roster should be composed of athlete leaders, with 19% occupying formal roles and 66% occupying informal roles. Furthermore, athletes reported that having an ideal number of athlete leaders created opportunities to share athlete leadership responsibilities and increased the resources available to the team. Moreover, an ideal number of athlete leaders was believed to positively influence a number of group dynamic constructs, including variables related to team structure (e.g., enhanced role clarity), team processes (e.g., team cohesion, collective efficacy), and outcomes (e.g., athlete satisfaction, performance; Crozier et al., 2013).

While Crozier et al. (2013) indicated that the presence of athlete leaders could potentially have a positive impact on many group dynamic variables, the current study concentrated on two specific group dynamic variables: cohesion and collective efficacy. The selection of cohesion in the current study was based on two premises. First, cohesion has long been considered one of the most important group variables in sport teams (Lott and Lott, 1965; Carron et al., 2002), meaning it is critical to team functioning (Carron et al., 1998). As such, cohesion is defined as “a dynamic process that is reflected in the tendency for a group to stick together and remain united in the pursuit of its instrumental objectives and/or for the satisfaction of member affective needs” (Carron et al., 1998, p. 213). One aspect of this definition is how cohesion reflects both a task and social orientation towards the group. Specifically, a task cohesion orientation refers to the general tendency of the group to stick together to achieve its objectives, while a social cohesion orientation represents feelings of closeness, similarity, and bonding of the group as a social unit.

Second, cohesion was selected for the current study given its positive association to athlete leadership behaviours. For example, Vincer and Loughead (2010) surveyed varsity athletes to examine the relationship between athlete leadership behaviours and perceptions of cohesion. Athlete leaders who were perceived as showing higher frequencies of the leadership behaviours of training and instruction (i.e., improving teammate performance) and social support (i.e., satisfying interpersonal needs of team members) had teammates with stronger perceptions of both task and social cohesion. Furthermore, the leadership behaviour of democratic behaviour (i.e., including group members in the decision process) was positively related to task cohesion. Similarly, Callow et al. (2009) found the athlete leadership behaviours of individual consideration (i.e., leaders attending to individual follower’s needs and concerns), fostering acceptance of group goals (i.e., leader behaviours that promote teamwork to achieve team goals), and high-performance expectations (i.e., leaders showing that he/she expects high standards from the team) were positively related to task cohesion. Additionally, fostering acceptance of group goals was positively associated with social cohesion.

In addition to cohesion, the other group dynamics variable targeted in the current study is collective efficacy. Bandura (1997) defined collective efficacy as a “group’s shared belief in its conjoint capability to organize and execute the courses of action required to produce given levels of attainment” (p. 477). Collective efficacy was selected for the current study due to its contribution to optimal team functioning, motivation, and perseverance, and its influence on individual team members’ behaviours, effort, and persistence in the face of adversity (Bandura, 1997). Similar to cohesion, collective efficacy has been shown to be related to athlete leadership. Specifically, Price and Weiss (2011) found that being viewed as an effective athlete leader was associated with teammates having greater perceptions of collective efficacy. Furthermore, athletes who rated themselves higher in athlete leadership behaviours reported greater collective efficacy.

With athlete leadership behaviours related to both cohesion and collective efficacy, it would be helpful to have a conceptual model that highlights these relationships. Frameworks used to study athlete leadership have largely been based on organizational psychology and sport coaching research. Two of the most widely used theoretical models are Chelladurai’s (2007) Multidimensional Model of Leadership (MML) and Avolio’s (1999) Full Range Model of Leadership (FRML). The MML is a procedural framework that assesses the relationship between constructs (e.g., athlete leadership and cohesion/collective efficacy), whereas the FRML is a behavioural framework assessing where behaviours fall on a continuum (i.e., passive versus active). The MML is a linear model composed of three factors: (a) antecedents, (b) leader behaviours, and (c) consequences. Antecedents consist of situational (i.e., team norms and goals), leader (i.e., leaders’ personal characteristics), and member characteristics (i.e., members’ personality, experience, and ability). Leader behaviours consist of three behaviour types: (a) required, (b) preferred, and (c) actual. Required behaviours refer to the types of behaviours the leader is expected to display. Preferred behaviours refer to the preferences of team members for certain leadership behaviours. The preferences for certain behaviours from the leader are determined by the team’s situation and the nature of the group. Actual behaviours refer to how the athlete leader behaves, and are largely dependent on the leader’s personal characteristics, such as personality, expertise, and experience. Finally, the consequences in the model refer to outcomes, such as performance and satisfaction. In relation to the present study, the model highlights that leadership behaviours can impact emergent team processes (e.g., cohesion and collective efficacy). Both these emergent team processes, cohesion (e.g., Grossman et al., 2021) and collective efficacy (e.g., Fuster-Parra et al., 2015), have an influence on performance. Consequently, it becomes important to develop these leadership behaviours in order to positively influence these emergent team processes.

The FRML proposes that effective leaders utilize a wide variety of behaviours including transformational leadership behaviours (Avolio, 1999). According to Bass and Riggio (2006), transformational leaders inspire their followers to commit to the team’s common goal and vision, challenge them to solve problems, and help them grow and develop into leaders themselves. The FRML highlights four different transformational leadership behaviours: a) idealized influence (i.e., leader sets a good example and instils pride), b) inspirational motivation (i.e., leader outlines a vision that is inspiring to followers), c) intellectual stimulation (i.e., leader challenges assumptions and encourages creativity), and d) individualized consideration (i.e., leader attends to individual follower’s needs and concerns). As such, leadership development involves enhancing the leadership capability by putting an emphasis on individual knowledge, skills, and abilities and by expanding the collective capacity of team members to engage effectively in leadership roles and processes (Day, 2001). Consequently, the MML and FRML were operationalized based on the leadership behaviours assessed in the the Leadership Scale for Sports (LSS; Chelladurai and Saleh, 1980) and the Differentiated Transformational Leadership Inventory (DTLI; Callow et al., 2009). These two inventories measure several different leadership behaviours. The assessment of these leadership behaviours is common in athlete leadership research (see Loughead, 2017 for a review).

Research focusing on athlete leadership development programs is limited. Currently, most athlete leadership development research has been conducted among two demographic groups, youth and intercollegiate athletes. Among the few studies focusing on youth athlete leadership development, Gould and Voelker (2010) found that their one-day workshop on how to be a high school team captain was helpful and enjoyable by the participants. Seeking to expand Gould and Voelker’s one-day workshop, Blanton et al. (2014) implemented a two-year long high school youth leadership club intended to develop leadership capabilities. The results indicated that the leadership development program was well received by the middle school students. At the intercollegiate level, Voight (2012) implemented a season-long athlete leadership development program with two NCAA Division I volleyball teams to improve team communication and team functioning and foster the personal leadership development of team captains. The captains reported the program had a positive impact on their personal leadership skills, team cohesion, and team and teammate performance. Expanding to include all athletes from two teams, Duguay et al. (2016) developed and administered a season-long athlete leadership development program. A total of 27 female varsity athletes participated in four 1 h-long leadership workshops throughout their season. The program positively impacted most of the athlete leadership behaviours targeted, specifically training and instruction, democratic behaviours, social support, positive feedback, appropriate role model, inspirational motivation, high performance expectations, and fostering acceptance of group goals and promoting teamwork. That is, participants reported employing these behaviours more after the leadership development program. Furthermore, the athlete leadership development program positively influenced athlete satisfaction and peer motivational climate (Duguay et al., 2016).

Although these studies highlight the benefits of conducting athlete leadership development programs, limitations remain. Primarily, Gould and Voelker (2010) and Voight (2012) simply stated their programs were grounded in leadership research and organizational psychology without any additional insight or information into the specific theories used to develop the leadership development program. Without a theoretical framework, a leadership development program can be nothing more than a collection of interesting leadership activities lacking an intentional and development approach (Redmond and Dolan, 2016). The current study attempted to fill this theoretical gap by conducting an athlete leadership development program grounded in the MML (Chelladurai, 2007) and the FRML (Avolio, 1999). Additionally, there were no quantitative measures used to objectively assess the results of these previous studies. The current study sought to address this gap including both quantitative measures and qualitative interviews through the use of a mixed methods approach. Youth sport was selected since the call for leadership development of young people is important for their social development (e.g., Wright and Côté, 2003). Youth who take on leadership roles are less likely to adopt negative bahviours (Allen et al., 2006). As such, we believe that leadership development for youth is a positive developmental activity that provides these individuals with supportive relationships (e.g., teammates) and opportunities to see themselves as having the ability to make valuable contributions to their team (e.g., leadership, cohesion, collective efficacy). Thus, the primary purpose of the current study was to implement and evaluate a theoretically-based leadership development program that targets the enhancement of athlete leadership behaviours, cohesion, and collective efficacy among male youth hockey players. Based on the success of Duguay et al.’s (2016) leadership development program among intercollegiate athletes, it was hypothesized that the athlete leadership development program would positively influence athlete leadership behaviours, cohesion, and collective efficacy of youth male hockey players.

## Materials and methods

### Participants

Participants in the current study were 14 male U17 ice hockey players from one Southwestern Ontario team playing in a competitive hockey league. In Canada, U17 is the second highest level of minor youth hockey. Players in the present study ranged in age from 15 to 17 years (*M* = 16.46, *SD* = 0.78) and had been playing hockey for an average of 10.79 years (*SD* = 2.04). The regular season for this team started in September and concluded in March. The team ended their season with a record of 3–26-5 (i.e., win-loss-tie), collecting 11 points out of a possible total of 68 points for a 16.18% winning percentage.

### Measures

#### Athlete leadership behaviours

Athlete leadership behaviours were assessed using two questionnaires. The first questionnaire was the Leadership Scale for Sports (LSS; Chelladurai and Saleh, 1980) consisting of 40 items and assessing five dimensions of leadership behaviours: training and instruction (13 items), positive feedback (5 items), social support (8 items), democratic behaviour (9 items), and autocratic behaviour (5 items). All responses on the LSS are scored on a 5-point Likert scale ranging from (1) *never* to (5) *always* with higher scores reflecting higher occurrences of the leadership behaviours. Vincer and Loughead (2010) found the LSS in measuring athlete leadership had a reasonably good model fit: CFI = 0.99, TLI = 0.98, and RMSEA = 0.05. Further, Cronbach’s alpha coefficients were as follows: training and instruction (α = 0.88), democratic behaviour (α = 0.79), autocratic behaviour (α = 0.74), social support (α = 0.86), and positive feedback (α = 0.84; Vincer and Loughead, 2010).

The second questionnaire used to measure athlete leadership behaviours was the Differentiated Transformational Leadership Inventory (DTLI; Callow et al., 2009). The DTLI contains 27 items and measures six transformational and one transactional behaviours: inspirational motivation (4 items), appropriate role modeling (4 items), individual consideration (4 items), intellectual stimulation (4 items), high performance expectations (4 items), fostering acceptance of group goals (3 items), and contingent reward (4 items). Each item from the inventory is scored on a 5-point Likert scale ranging from (1) *not at all to* (5) *all the time.* Callow et al. (2009) found a very good fit for this 6-factor model: CFI = 0.98, SRMR = 0.06, NNFI = 0.98, and RMSEA = 0.05. Further, Cronbach’s alpha coefficients were as follows: individual consideration (α = 0.66), fostering acceptance of group goals (α = 0.73), high performance expectations (α = 0.86), appropriate role model (α = 0.81), inspirational motivation (α = 0.75), intellectual stimulation (α = 82), and contingent reward (α = 0.82) (Callow et al., 2009).

#### Cohesion

Cohesion was assessed using the Youth Sport Environment Questionnaire (YSEQ; Eys et al., 2009). The YSEQ was developed to measure cohesion in adolescent athletes aged 13–17 years. The YSEQ is a 16-item questionnaire measuring task (8 items) and social cohesion (8 items). All items are scored on a 9-point Likert scale ranging from (1) *strongly disagree* to (9) *strongly agree*, with higher scores reflecting greater perceptions of cohesion. Confirmatory factor analyses provided support for the factorial validity of the YSEQ with an acceptable model fit: CFI = 0.90 and SRMR = 0.07 (Eys et al., 2009). Further, Cronbach’s alpha coefficients were as follows: task cohesion (α = 0.92) and social cohesion (α = 0.94) (Bruner et al., 2014).

#### Collective efficacy

Players’ perceptions of their team’s collective efficacy were assessed using the Collective Efficacy Questionnaire for Sports (CEQS; Short et al., 2005). The CEQS is a 20-item questionnaire that measures the five dimensions of collective efficacy: ability (4 items), effort (4 items), persistence (4 items), preparation (4 items), and unity (4 items). All items are scored on an 11-point Likert scale, ranging from (0) *not at all confident* to (10) *extremely confident*, with higher values representing a greater rating of the team’s confidence in their ability to successfully achieve a goal. A CFA revealed a good model fit: CFI = 0.92, NNFI = 0.90, SRMR = 0.04, and RMSEA = 0.09 (Short et al., 2005). Further, Cronbach’s alpha coefficients were as follows: ability (α = 0.91), effort (α = 0.87), persistence (α = 0.81), preparation (α = 0.87), and unity (α = 0.85) (Short et al., 2005). A correlation matrix showing the relationship between collective efficacy and cohesion dimensions are found in Table 1.

**Table 1:** Intercorrlations between task cohesion, social cohesion, and collective efficacy.

Dimension	Pre-intervention							Post-intervention						
	Task	Social	Ability	Effort	Pers.	Prep.	Unity	Task	Social	Ability	Effort	Pers.	Prep.	Unity
Pre-intervention														
Cohesion														
Task	1													
Social	0.21	1												
Collective Efficacy														
Ability	0.57^*^	0.26	1											
Effort	0.44	0.37	0.60^**^	1										
Pers.	0.76^**^	0.18	0.82^***^	0.70^**^	1									
Prep.	0.74^**^	−0.02	0.73^**^	0.58^*^	0.85^***^	1								
Unity	0.81^***^	0.18	0.81^***^	0.74^**^	0.85^***^	0.81^***^	1							
Post-intervention														
Cohesion														
Task	0.38	0.19	0.35	0.52^**^	0.55^*^	0.57^*^	0.39	1						
Social	0.43	0.84^***^	0.46	0.27	0.28	0.25	0.31	0.19	1					
Collective efficacy														
Ability	0.35	−0.06	0.77^***^	0.72^**^	0.72^**^	0.62^*^	0.67^*^	0.56^*^	0.03	1				
Effort	0.44	−0.18	0.53^*^	0.60^*^	0.74^**^	0.69^**^	0.55^*^	0.75^**^	−0.14	0.79^***^	1			
Pers.	0.45	−0.07	0.56^*^	0.69^**^	0.63^**^	0.59^*^	0.60^*^	0.67^**^	−0.01	0.80^***^	0.88^***^	1		
Prep.	0.37	−0.17	0.52	0.63^*^	0.64^**^	0.60^*^	0.58^*^	0.76^**^	−0.12	0.74^**^	0.87^***^	0.91^***^	1	
Unity	0.35	−0.07	0.68^**^	0.69^**^	0.71^**^	0.68^**^	0.65^**^	0.76^**^	−0.02	0.93^***^	0.84^***^	0.79^***^	0.86^***^	1

Pers., persistence; Prep., preparation.

* *p* < 0.05;

** *p* < 0.01;

*** *p* < 0.001.

### Procedure

Prior to data collection, ethics approval was obtained from the authors’ university ethics board. Data collection occurred at two-time points: baseline (i.e., beginning of the season prior to the leadership development intervention) and post-intervention (i.e., end of the season). At baseline, athletes were asked to read and sign a consent to participate in research form. Pre-intervention questionnaires were administered measuring demographics, athlete leadership behaviours (i.e., LSS, DTLI), cohesion (i.e., YSEQ), and collective efficacy (i.e., CEQS). Following baseline testing, athletes participated in six leadership development workshops, approximately every 3 weeks, over the course of the season. The baseline data collection occurred at the end of October and post-intervention data were collected at the end of January. Each workshop lasted approximately 45–60 min. One week following the final workshops, participants completed all of the questionnaires post-intervention. One month following post-intervention data collection, a focus-group interview was conducted to evaluate the impact of the athlete leadership development program.

### Athlete leadership intervention

The six workshops were based on Duguay et al.’s (2016) athlete leadership development program. The athlete leadership development program was theoretically grounded using both Chelladurai’s (2007) MML and Avolio’s (1999) FRML frameworks. The MML was used to guide the development of the content of the workshops. In particular, the model states that aspects such as age of the athletes should be taken into consideration; therefore it was important to have age appropriate language and examples when teaching the leadership behaviours during the intervention. Further, the MML also hypothesizes that the leadership behaviours influence team outcomes and in this case we would discuss how the leadership behaviours could influence aspects such as cohesion and collective efficacy. As for the FRML, this model states that effective leaders utilize numerous and a wide host of leadership behaviours. Empirical support for this premise was found by Duguay et al. (2016) indicating that it is important for athletes to utilize a wide range of leadership behaviours (see Table 2 for a list of those leadership behaviours).

**Table 2:** Workshop behaviours and sample activities.

Behaviours and sample activities
**Leadership behaviours**
Training and instruction • Leader behaviours aimed at improving the athlete’s performance through physical and skill development - Athletes reflect on their technical, tactical, physical, and mental skills
Democratic behaviour • Leader involves his/her teammates in the decision-making process - Ahletes reflect on how they could encourage inclusive decision making on their team
Social support • Show concern for teammates’ welfare by establishing warm interpersonal relationships - Athletes given three cases and explore options for providing social support
Positive feedback • Reinforce teammates by recognizing and rewarding good performance - Athletes share influential positive feedback they have received and explain what made it effective
Individual consideration • Leaders are empathetic, supportive, and attends to individual follower’s needs and concerns. - Athletes reflect on how they could pay more attention and show respect for each teammate
Inspirational motivation • Leaders developing, articulating, and inspiring teammates with their vision for the future - Athletes discuss the motivational effects of a sports video clip by reflecting on how they become motivated to perform their best
Intellectual stimulation • Leaders challenge assumptions, encourage their followers to be creative, and are open to new ways to solve problems. - Athletes examine how their team has handled various roadblocks and whether they could have been handled differently
Acceptance of group goals • Leader behaviours that promote teamwork to achieve team goals. - Athletes make a link between their individual goals and their team goals
High performance expectations • Leaders showing that they have high standards for the team - Athletes explore the expectations they hold for themselves and their teammates
Appropriate role model • Set examples for teammates that are consistent with the values the team promotes - Athletes reflect on how they be a role model on their team **Outcomes** Task cohesion • Tendency of the group to stick together to achieve its objectives - Athletes work together to build the talled tower with marshmallow and spaghetti Social cohesion • Feelings of closeness, similarity, and bonding of the group as a social unit - Athletes form a circle and lock arms and work together to untangle themselves Collective efficacy • Team’s confidence in their ability to achieve their goals - Athletes build launching machine to throw cotton balls

Bulleted points are the athlete leadership behavioural principles and dashes represent athlete leadership development sample activities.

As for the delivery of the workshops, we utilized an educational approach recommended by Whetten and Cameron (2011), whereby the participants were given (1) a presentation of the leadership behaviours to be learned, (2) a demonstration of the leadership behaviours in action, (3) the opportunity to practice these newly learned leadership behaviours, and (4) feedback from peers and the instructor. The explanation of the leadership behaviours was accompanied by examples of appropriate and inappropriate applications, in addition to an analysis of why and how they can be effective or ineffective. In addition, participants worked either individually or in small groups to complete activities designed to reinforce and practice the leadership behaviours covered within each workshop, receiving feedback and assistance at every step along the way from peers and the instructor (i.e., first author). All activities finished with a team discussion highlighting how these leadership behaviours benefited the participants themselves and the team as a whole. Finally, participants were encouraged to apply and foster the development of these leadership behaviours to their sport of ice hockey. An outline of the leadership behaviours covered in each workshop is provided in Table 2. To encourage maximum participation from the participants, workshops were delivered prior to the team’s practices. Consequently, nearly every participant was present for each workshop. The four absences by four separate players occurred due to a work conflict, school commitment, or illness. The participants were provided with a leadership workbook to support, reinforce, and expand on the material presented in the workshops that they could refer to outside of the workshop sessions. The workbook included an introduction to the importance of leadership development, important terms and definitions, activities to accompany the topics (i.e., leadership, cohesion, collective efficacy), and a reflection section.

### Data analyses

#### Quantitative analysis

The data were screened for missing values, outliers, skewness, and kurtosis. The data were deemed to be normally distributed, therefore no transformations to the data were necessary. To determine whether there were differences in leadership behaviours, cohesion, and collective efficacy from pre-to post-intervention, a series of Bayesian paired-samples *t* tests were carried out to determine the impact of the intervention.

Calculating Bayes factors provides evidence in favour of the null hypothesis or the alternative hypothesis (Wagenmakers, 2007). In contrast to frequentist *p* values, Bayes factors provide a more accurate estimate of the evidence present in the data available (Wagenmakers, 2007). In Bayesian analyses, the posterior distribution (equivalent to Frequentist point estimates and standard error) is a combination of prior distributions (determined by the researcher) and the likelihood (determined by the data). An advantage of Bayesian analyses is the inclusion of prior information into the model through prior distributions, which can help the accuracy of predictions (McNeish, 2016). The inclusion of prior distributions in the analysis allows research with small sample sizes to base the results on more information than is available from the data itself. The contribution of the prior distribution and likelihood to the posterior distribution is not equal. When dealing with a small sample size, the prior distribution is given more weight than the likelihood (McNeish, 2016).

For the current study, the weight of the athlete leadership and cohesion priors were set based on the results of Duguay et al.’s (2016) study (i.e., Cohen’s d and standard deviation). Due to the lack of prior knowledge regarding the relationship between collective efficacy and athlete leadership, the weight of the prior for collective efficacy was set at the default value of 0.707, which is the recommended weighting when no prior information is known (Hoffmann, 2019).

Bayesian analyses were conducted using the JASP software (JASP Team, 2018). The results from Bayesian analyses are reported in the form of a Bayes factor. In particular, a Bayes factor of BF_+0_ quantifies evidence for the one-sided alternative hypothesis (H_1_) that the difference is larger than zero. Additionally, a Bayes factor of BF_0+_ quantifies evidence for the null hypothesis (H_0_) relative to the one-sided alternative hypothesis that the difference is larger than zero. According to Jeffreys (1961), Bayes factors below 1 represent weak evidence, Bayes factors between 1 and 3 represent anecdotal evidence, Bayes factors between 3 and 10 represent substantial evidence, Bayes factors between 10 and 30 represent strong evidence, Bayes factors between 30 and 100 represent very strong evidence, and Bayes factors above 100 represent decisive evidence.

#### Qualitative analysis

Following the end of the intervention, an email was sent to all participants asking if they wanted to participate in a focus group. The four athletes who responded were members of the team’s leadership group consisting of one captain and three assistant captains. The purpose of this focus group was to allow participants to reflect on their season and qualitatively evaluate the effectiveness of the athlete leadership development program. Athletes were able to provide a detailed account of their personal opinions and perceptions concerning the leadership program and its effect on individual players and the team as a whole.

The qualitative aspect of this study was conducted using a constructivist philosophical position, focusing on understanding the meanings people create for themselves and attribute to their experiences (Tamminen and Poucher, 2020). The underlying assumptions of constructivism include a relativist ontology and a subjectivist and transactional epistemology. According to the relativist ontological position, there is no single external reality independent of the individual, that is, reality exists in the form of multiple individual constructions about the world shaped through lived experiences (Tamminen and Poucher, 2020). Essentially, a relativist viewpoint implies that different people will make different interpretations of their experiences. Therefore, the purpose of conducting qualitative research from a relativist ontological position is to attempt to understand the various interpretations people make about their experiences, and to try and understand why people view things the way they do (Tamminen and Poucher, 2020).

To better understand the various idiosyncrasies concerning people’s experiences, the constructivist viewpoint assumes a subjectivist and transactional epistemological position (Tamminen and Poucher, 2020). That is, knowledge is created through transactions between the researcher and participant (e.g., focus group interview), and the researcher cannot separate themselves from their previous experiences and their interpretations of those experiences. In fact, the researcher’s subjective understandings about a phenomenon or experience cannot be removed from the research process and/or findings (Tamminen and Poucher, 2020). As such, meaning and knowledge are created based on interdependent interactions between individuals. This notion of a subjectivist and transactional epistemology underlies the concept of co-construction of knowledge. That is, both the participant and the researcher bring their own understandings about the meanings of experiences to their interactions. Essentially, during an interview, the researcher forms interpretations and meanings concerning the participant’s interpretations of an experience (Tamminen and Poucher, 2020).

The data were examined using hierarchical content analysis, allowing for the identification and description of patterns in the data (Sparkes and Smith, 2014). Specifically, meaningful pieces in the transcript were organized into raw data themes. Next, themes that appeared to fit well together were combined into categories. Athletes’ names were changed for the quotes below to HP (i.e., hockey player) and a given a number (e.g., HP1). The study followed Sparkes and Smith’s (2014) concept of reflexivity and Smith and McGannon’s (2018) recommendations to utilize member reflections and critical friends. The second author, who has extensive experience in conducting leadership development programs and leading focus groups, served as a critical friend and met with the lead author at every step of the analysis to promote reflexivity and explore various interpretations of the data.

An interview guide, composed of four sections, was developed for this stuy and is available upon request. The interview guide consisted of questions designed to create discussion around the team’s performance throughout the season, athlete’s impressions of the leadership development program, the content of the responses, and concluding questions that allowed participants to provide recommendations on how to improve the athlete leadership development program. The focus group was audio recorded and transcribed verbatim.

## Results

### Descriptive statistics

Means, standard deviations, and internal consistencies for the leadership behaviours, cohesion, and collective efficacy are presented in Table 3. For the athlete leadership behaviours, the means for social support, positive feedback, inspirational motivation, intellectual stimulation, acceptance of group goals, high performance expectations, appropriate role model, and contingent reward trended downward from pre-to post-intervention, while democratic behaviour, autocratic behaviour, and individual consideration trended upward. Training and instruction remained the same from pre-to post-intervention. As for cohesion and collective efficacy, both the means of task and social cohesion trended upward from pre-to post-intervention, while the means for the five dimensions of collective efficacy trended downward.

**Table 3:** Descriptive statistics for athlete leadership behaviours, cohesion, and collective efficacy.

Variable	Pre-intervention			Post-intervention		
	*M*	*SD*	*α*	*M*	*SD*	*α*
*Leadership*						
TI	3.24	0.61	0.87	3.24	0.62	0.83
DB	3.59	0.76	0.75	3.61	0.83	0.88
AB	2.53	0.83	0.81	2.86	0.83	0.71
SS	3.96	0.58	0.77	3.74	0.59	0.71
PF	4.57	0.48	0.85	4.11	0.76	0.79
IC	3.95	0.84	0.59	3.98	0.65	0.83
IM	4.04	0.60	0.76	3.83	0.69	0.62
IS	3.57	0.66	0.85	3.26	0.96	0.90
AGG	4.24	0.62	0.78	3.83	0.60	0.61
HPE	4.20	0.61	0.66	4.14	0.53	0.59
ARM	4.06	0.61	0.77	3.94	0.77	0.87
CR	4.29	0.49	0.79	4.11	0.58	0.83
*Cohesion*						
Task	6.46	1.09	0.85	6.54	1.55	0.93
Social	6.97	1.98	0.96	6.98	1.73	0.94
*Collective efficacy*						
Ability	6.95	1.94	0.85	6.77	1.61	0.86
Effort	7.73	1.35	0.77	7.02	1.75	0.83
Persistence	7.27	1.65	0.82	6.99	1.71	0.90
Preparation	7.57	1.69	0.90	7.23	2.03	0.92
Unity	7.61	1.36	0.88	7.52	1.37	0.75

Scores for the leadership behaviours range from 1 to 5, Cohesion from 1 to 9, and Collective efficacy from 1 to 10. TI, Training and Instruction; DB, Democratic Behaviour; Autocratic Behaviours; SS, Social Support; PF, Positive Feedback; IC, Individual Consideration; IM, Inspirational Motivation; IS, Inspirational Motivation; AGG, Fostering Acceptance of Group Goals and Promoting Teamwork; HPE, High Performance Expectations; ARM, Appropriate Role Model; CR, Contingent Reward.

### Quantitative analysis

#### Athlete leadership behaviours

When quantifying evidence in favour of the alternative hypothesis (H_1_), the Bayesian paired samples *t* tests indicated weak evidence for training and instruction (BF_+0_ = 0.12), democratic behaviour (BF_+0_ = 0.11), social support (BF_+0_ = 0.55), appropriate role model (BF_+0_ = 0.34), inspirational motivation (BF_+0_ = 0.61), high performance expectation (BF_+0_ = 0.31), intellectual stimulation (BF_+0_ = 0.72), and individual consideration (BF_+0_ = 0.27). Additionally, substantial evidence was found for positive feedback (BF_+0_ = 3.18) and acceptance of group goals (BF_+0_ = 3.00).

When quantifying evidence in favour of the null hypothesis (H_0_), the Bayesian paired samples *t* tests indicated weak evidence for positive feedback (BF_0+_ = 0.32) and fostering acceptance of group goals (BF_0+_ = 0.34). However, the results indicated anecdotal evidence for social support (BF_0+_ = 1.82), inspirational motivation (BF_0+_ = 1.66), and intellectual stimulation (BF_0+_ = 1.39). Finally, the results indicated substantial evidence for training and instruction (BF_0+_ = 8.06), democratic behaviour (BF_0+_ = 4.27), appropriate role model (BF_0+_ = 3.00), high performance expectation (BF_0+_ = 3.25), and individual consideration (BF_0+_ = 3.68).

#### Cohesion

When quantifying evidence in favour of the alternative hypothesis (H_1_), the Bayesian paired samples *t* tests indicated weak evidence for both task cohesion (BF_+0_ = 0.09) and social cohesion (BF_+0_ = 0.15). When quantifying evidence in favour of the null hypothesis (H_0_), the Bayesian paired samples *t* tests indicated substantial evidence for social cohesion (BF_0+_ = 6.48) and strong evidence for task cohesion (BF_0+_ = 10.65).

#### Collective efficacy

When quantifying evidence in favour of the alternative hypothesis (H_1_), the Bayesian paired samples *t* tests indicated weak evidence for ability (BF_+0_ = 0.42), persistence (BF_+0_ = 0.50), preparation (BF_+0_ = 0.52), and unity (BF_+0_ = 0.34), and anecdotal evidence for effort (BF_+0_ = 2.00). When quantifying evidence in favour of the null hypothesis (H_0_), the Bayesian paired samples *t* tests indicated weak evidence for effort (BF_0+_ = 0.50), anecdotal evidence for ability (BF_0+_ = 2.34), persistence (BF_0+_ = 2.00), and preparation (BF_0+_ = 1.93), and substantial evidence for unity (BF_0+_ = 3.00).

### Qualitative analysis

A focus-group interview was conducted to evaluate the impact of the athlete leadership development program with four members of the ice hockey team. These participants consisted of the team’s leadership group. Based on the focus group interview, athletes’ responses were group into four themes focused on cohesion, communication, shared leadership, and the benefits of the leadership program.

#### Cohesion

The participants described some of the ways the athlete leadership development program positively influenced cohesion. In particular, they described how the leadership program was able to bring them closer together as a team.

It [athlete leadership development program] brought us together more, we all got along before, but we were not really united. It brought us all together as more than just friends. If something happened on the ice, everyone took it to heart. For instance, if someone got checked from behind or got high-sticked and got hurt, everyone took it to heart. We just cared about each other more (HP1).

Furthermore, the athlete leadership development program was useful in maintaining the team’s cohesiveness despite having a lack of team success. As HP2 noted:

The leadership program gave us the mindset that hockey is a team sport and while losing is difficult, the program put it into perspective that you have to stick together win or lose. Our win-loss record definitely does not imply that we had a good season, but we bonded as a team, we got closer and this was important since we had a lot of new players this year on the team.

Despite not having a successful season, the participants discussed how the leadership behaviors they learned during the workshops impacted the way the team played. In particular, the participants noted that the team played in a tournament following the season and credited the way the team performed well to what they learned throughout the workshops. Specifically, the players mentioned being more cohesive, which influenced their performance.

The team benefited from it [the leadership program] because right after the season, we had a tournament and we won most of our games, we only lost one game. We got a lot of goals but the leadership program inspired everyone to work together and be on the same page (HP3).

#### Communication

The players mentioned how the athlete leadership development program helped them deal with their frustrations (e.g., losing games) by teaching them to communicate more effectively with one another. As one participant noted, “We were talking to each other more, people were actually stepping up and saying what they had to say” (HP4). The enhanced communication was particularly useful in dealing with conflicts that occurred throughout the season.

When conflict arose, instead of yelling and getting mad at each other, we just told ourselves let us settle down, talk it out, and find a good solution that benefits both sides and let us get back in the game and focus (HP3).

#### Shared leadership

The participants noted how the athlete leadership development program taught them about the importance of sharing the leadership responsibilities among team members. As one player noted,

I learned that you do not have to have a “C” or an “A” on your jersey to be a leader. Anyone can step up. As well, you do not have to necessarily be a verbal leader, you can lead by example (HP2).

The participants also expressed how their own leadership behaviours impacted their teammates, “The athlete leadership development program taught you how to make everyone around you a leader as well and teach everyone else how to lead the team” (HP3).

#### Benefits of the leadership program

The participants revealed some of benefits of the athlete leadership development program. Specifically, the participants discussed learning how to motivate their teammates and taking their teammates’ opinion into consideration when making decisions. One player noted,

The athlete leadership development program taught me how to motivate my teammates, get them to be on the same page, be more open minded. It also taught me how to take other people’s opinion and work it in with my own ideas and form one single plan that would work for everyone (HP1).

The participants also discussed how the leadership program was useful outside of hockey. The players noted that they transferred the knowledge gained from this program to other aspects of their life.

I used what I learned here and brought it to the classroom. For instance, at school one thing we have to do is to help younger students with their studies. So, I definitely used these skills and transferred them over to different aspects of my life. (HP2).

## Discussion

The purpose of the current study was to implement and evaluate an athlete leadership development program targeting the enhancement of athlete leadership behaviours, cohesion, and collective efficacy with male youth hockey players from one team. It was hypothesized that the athlete leadership development program would positively impact athlete leadership behaviours, cohesion, and collective efficacy. The results partially support this hypothesis. When quantifying evidence in favour of the alternative hypothesis, the results of the Bayesian paired-samples *t* tests indicated weak to anecdotal evidence. That is, the athlete leadership development program did not positively impact the measured constructs pre-to post-intervention. However, when quantifying evidence in favour of the null hypothesis of no change, the results indicated that the program maintained the level of athlete leadership behaviours, cohesion, and collective efficacy throughout the season. These findings are corroborated in the focus group interview which showed that the athlete leadership development program was beneficial in helping the players maintain their leadership behaviours, along with perceptions of cohesion and collective efficacy. Further benefits from the focus group interview included better communication amongst team members and dealing with conflict more effectively.

Beyond these findings, one aspect of the current study that should be kept in mind when interpreting the results is the team’s performance throughout the season. The team finished their season with a record of 3–26-5 (i.e., win-loss-tie), collecting 11 points out of a possible total of 68 points for a 16.18% winning percentage. When we started delivering our athlete leadership development program with this team, we did not know that their on-ice performance was going to be poor as the season progressed. Very little research has examined the impact of losing within the context of sport teams. However, as Van Puyenbroeck et al. (2019) noted, losing games negatively impacts a group’s dynamics. One way that losing may impact a group’s dynamics is through the “bad apple phenomenon” (Felps et al., 2006), whereby negative group members can have repercussion on team functioning. In sport, these types of athletes are labeled *cancers* (Cope et al., 2010). As for the consequences of team cancers, these athletes can become a distraction to other team members, engage in negative behaviours that affect the team, form cliques that are destructive to team functioning, and decrease a team’s cohesiveness (Cope et al., 2010). We believe that our athlete leadership development program was able to mitigate the emergence of team cancers on this losing team and the associated negative consequences.

The quantitative results indicated the participants did not increase their use of athlete leadership behaviours following the athlete leadership development program, as measured using the LSS (Chelladurai and Saleh, 1980) and the DTLI (Callow et al., 2009). This result may have been due to the high baseline mean scores of the athlete leadership prior to the start of the intervention. In Duguay et al. (2016), the authors measured athlete leadership behaviours using both the LSS and DTLI using a pre-and post-intervention design. Duguay et al. (2016) reported post-intervention mean scores for the athlete leadership behaviours that are similar or lower than the pre-intervention mean scores from the current study. As such, it may have been difficult for participants in the current study to increase their scores significantly post-intervention. That is, participants in the current study reported using leadership behaviours to a high degree at the beginning of the athlete leadership development program. Therefore, when the athletes completed the post-intervention leadership behaviour questionnaires, it would have been difficult for the mean scores to increase greater than what was reported at baseline.

In fact, the findings of the focus group interview helped shed light on the usefulness of the intervention on the development of various leadership behaviours. The participants mentioned learning how to step up and take action, regardless of whether they fulfilled a formal leadership role (e.g., team captain). Additionally, participants mentioned learning how to communicate more effectively with teammates, remain positive in face of conflict, share the leadership roles, and the importance of staying cohesive. This is consistent with previous athlete leadership development research where participants in Duguay et al. (2016) mentioned that the leadership development program encouraged team members to step up and fulfill leadership roles. Similarly, athletes in Voight’s (2012) study reported that the leadership program taught them what it takes to be a leader, and how to effectively communicate with teammates. Players in the current study also mentioned that the program helped put their performance-related frustrations from losing regularly into perspective, emphasizing that hockey is a team sport and as such the team must work together to overcome these frustrations.

Similar to the athlete leadership behaviours, cohesion levels also were maintained from pre-to post-intervention. This result is similar to the findings of Senécal et al.’s (2008) study examining the effects of a season-long team-building intervention aimed at enhancing cohesion. Athletes in the intervention group showed no significant increase in cohesion from the beginning of the season to the end of the season, while athletes in the control condition showed a significant decrease in cohesion during the season. Based on their study (i.e., Senécal et al., 2008), it would appear that the intervention was helpful in maintaining levels of cohesion throughout the season. This result is impressive when examining research that has measured cohesion over the course of a season with no intervention. That is, researchers have reported decreases in levels of cohesion over the course of a season (Heuzé et al., 2006, 2007; Leo et al., 2012). In their investigation of basketball and handball perceptions of cohesion, Heuzé et al. (2006) found that players reported higher levels of cohesion at the beginning of the season. However, over the course of the season, players reported lower levels of cohesion. As mentioned in Carron et al.’s (1998) definition, cohesion is a dynamic process where changes in cohesion is impacted by a wide variety of personal, environmental, and team factors (Carron et al., 2002). In the context of the present study, one team factor that may have played a role is the team’s poor performance throughout the season. Nonetheless, it should also be noted that the mean scores of task cohesion at baseline (i.e., *M* = 6.46) in the current study was similar to the level of task cohesion in previous research utilizing the YSEQ (e.g., Bruner et al., 2014, *M =* 6.66; McLaren et al., 2015, *M =* 7.01; Vierimaa et al., 2018, *M =* 5.99). In contrast, the mean score for social cohesion in the current study (*M =* 6.97) was higher than in other studies (e.g., Bruner et al., 2014, *M =* 6.27; McLaren et al., 2015, *M =* 4.02). As such, it appears that the athletes in the current study were already a fairly cohesive team at baseline. It is possible that a comparable effect to Senécal and colleagues occurred in the present study given the results of Bayesian *t* tests when quantifying evidence in favour of the null hypothesis of no change. However, it is difficult to ascertain this without the presence of a control condition.

The results of the current study are impressive when you consider the losing record of the team. Meta-analyses examining cohesion (Carron et al., 2002; Grossman et al., 2021) have found that this construct has a moderate to large positive impact on performance, indicating that higher levels of cohesion are associated with more successful performances in various sports (Muthiane et al., 2015). In fact, perceptions of cohesion are significantly lower for athletes on losing teams than those on winning teams (Muthiane et al., 2015). When asked in the focus group interview concerning the cohesiveness amongst teammates, the participants mentioned that the leadership program brought the team closer together and made them feel more united, despite many team members already being friends and getting along before the start of the program.

Conflict and communication were two unique results from the focus group interview pertaining to the benefits of having participated in the athlete leadership development program. Athletes noted their poor on-ice performances created moments of conflict amongst the team. However, the participants mentioned that the leadership development program provided them with the skills to communicate more effectively, allowing them to constructively deal with conflict by finding solutions that benefited all team members. According to Dionne et al. (2004), communication and conflict management are crucial processes to team development. Moreover, communication is an essential component in preventing, processing, and resolving conflicts (Rhind and Jowett, 2010). As such, athletes who accept each other and deal with disputes in constructive and integrative ways are better equipped at managing conflict (Sullivan and Feltz, 2003). Consequently, the athlete leadership development program in the current study may have provided the participants with the necessary skills to more effectively deal with their intra-team conflicts, especially during a losing season.

Additionally, participants mentioned utilizing the skills they learned during the leadership development program outside of the sporting context. The findings of the current study are consistent with previous research exploring life skill development and transfer among wrestlers (Pierce et al., 2016), where several participants reported applying the skills learned outside their sport, including leadership (Pierce et al., 2016). However, it is important to consider the fact that the transfer of skills is not a guaranteed outcome of the learning process. In fact, mere participation in sport does not guarantee a transfer of the skills acquired through sport (Trottier and Robitaille, 2014).

The current study is not without limitations. First, the small sample size may have impacted the statistical power. Studies with low statistical power have a reduced ability to detect a true effect (Button et al., 2013). Therefore, it is important for future researchers to recruit more participants to further examine the impact of an athlete leadership development program. Second, without the inclusion of a control group, it is difficult to determine with certainty whether the intervention was effective at maintaining the levels of athlete leadership behaviours, cohesion, and collective efficacy, which in turn helped buffer the effect of losing. Another possible limitation to this study is the athlete leadership inventories used in this study (i.e., LSS and DTLI). These inventories were developed for adult populations and primarily utilized with intercollegiate athletes. Paradis and Loughead (2009) presented the factorial validity of the LSS and DTLI for youth populations. The results revealed good factorial validity for both inventories. Further, an application of the Flesch–Kincaid assessment of readability to the items contained within the LSS and DTLI resulted in a sixth-grade level of readability (i.e., youth aged 12–13). Therefore, we felt confident that the LSS and DTLI could be used with 17-year-old participants. Consequently, the development of a youth athlete leadership inventory may by useful to more accurately capture the factors that are important to younger athletes. It is worth noting that for the qualitative focus group interview, the participants consisted of the team’s leadership group. While all of the participants were invited, only the team’s leadership group volunteered for this aspect of the study. Having participants who were involved in the team’s formal leadership structure raises questions pertaining to homogenous sampling. Future research should strive to include a diversity of participant including those not holding a formal leadership role within the team. Also, emanating from the interview was the emergence of enhanced communication and conflict resolution as a result of the athlete leadership development program. Unfortunately, we did not collect quantitative data on these two outcomes. Future research should examine other outcomes of the leadership program.

Taken together, the results from the current study provide researchers, coaches, and mental performance consultants with preliminary evidence highlighting the importance of an athlete leadership development program as a method of maintaining levels of athlete leadership behaviours, cohesion, and collective efficacy throughout the season. Specifically, is appears that the athlete leadership development program can potentially act as a buffer against the negative effects of poor performance. Hopefully, this study will lead to further examination into the benefits of developing athlete leadership behaviours, cohesion, and collective efficacy. Finally, it is hoped that the information presented in this study will encourage coaches and mental performance consultants to implement an athlete leadership development program with their teams.

## Data availability statement

The raw data supporting the conclusions of this article will be made available by the authors, without undue reservation.

## Ethics statement

The studies involving human participants were reviewed and approved by University of Windsor Research Ethics Board. Written informed consent from the participants’ legal guardian/next of kin was not required to participate in this study in accordance with the national legislation and the institutional requirements.

## Author contributions

MB implemented the athlete leadership development program, analyzed the data, and wrote the first draft of the manuscript. TL and KM-C contributed to the development of the athlete leadership development program, assisted in analyzing the data, and writing/editing subsequent drafts of the manuscript. All authors contributed to the article and approved the submitted version.

## Funding

This research was supported by the Social Sciences and Humanities Research Council (SSHRC) Insight Grant program (grant no. 435-2020-0526).

## Conflict of interest

The authors declare that the research was conducted in the absence of any commercial or financial relationships that could be construed as a potential conflict of interest.

## Publisher’s note

All claims expressed in this article are solely those of the authors and do not necessarily represent those of their affiliated organizations, or those of the publisher, the editors and the reviewers. Any product that may be evaluated in this article, or claim that may be made by its manufacturer, is not guaranteed or endorsed by the publisher.
